# Risk Factors for Clinically Negative Level II Cervical Lymph Node Metastasis in Papillary Thyroid Carcinoma

**DOI:** 10.3390/jcm14176217

**Published:** 2025-09-03

**Authors:** Dongju Kim, Seunguk Bang, Gwangju Yu

**Affiliations:** 1 Department of Surgery, Daejeon St. Mary’s Hospital, College of Medicine, The Catholic University of Korea, Seoul 03083, Republic of Korea; reallydong@gmail.com; 2 Department of Anesthesiology and Pain Medicine, Daejeon St. Mary’s Hospital, College of Medicine, The Catholic University of Korea, Seoul 03083, Republic of Korea; djgass@hanmail.net

**Keywords:** lymphatic metastasis, neck dissection, occult level II metastasis, papillary thyroid carcinoma, risk factor, thyroid neoplasm, ultrasonography

## Abstract

**Objectives**: Papillary thyroid carcinoma (PTC) frequently presents with cervical lymph node metastasis, even in small tumors, and lateral lymph node involvement serves as an important prognostic factor. Therapeutic lateral neck dissection is typically recommended when nodal metastasis is clinically evident, usually including levels II–V. However, the necessity of routine level II dissection in patients without clinical or radiologic evidence of level II involvement remains controversial, given its association with increased surgical morbidity, particularly injury to the spinal accessory nerve. Identifying reliable clinicopathological predictors of occult level II metastasis may enable more selective surgical approaches that minimize unnecessary dissection while preserving oncologic safety. Therefore, this study aimed to identify clinicopathological risk factors associated with occult level II lymph node metastasis in patients with PTC who have clinically positive lateral nodes but no clinical evidence of level II involvement. **Methods**: We retrospectively analyzed 1247 patients who underwent thyroidectomy for PTC between 2015 and 2022. Of these, 67 patients with clinically positive lateral lymph node metastasis and clinically negative Level II nodes who underwent therapeutic lateral neck dissection were included. Clinicopathological features were compared between patients with and without occult Level II metastasis. Univariate and multivariate logistic regression analyses were performed to identify independent risk factors. **Results**: Among the 67 patients analyzed, 24 (35.8%) had occult Level II metastasis. Compared to those without, patients with occult Level II metastasis had significantly larger primary tumors (2.18 ± 1.31 cm vs. 1.51 ± 1.02 cm, *p* = 0.024), a greater number of central lymph node metastases (5.88 ± 4.41 vs. 3.37 ± 2.66, *p* = 0.005), larger maximum size of metastatic central lymph node (1.44 ± 1.07 cm vs. 0.87 ± 0.48 cm, *p* = 0.004), and a higher number of metastatic lateral lymph nodes (7.63 ± 3.75 vs. 3.19 ± 2.21, *p* < 0.001). Multivariate analysis identified the number of metastatic lateral lymph node as the only independent predictor of occult Level II involvement (OR = 1.57, 95% CI: 1.213–2.044, *p* = 0.001). The final multivariate model demonstrated a Nagelkerke R^2^ of 0.46. ROC curve analysis confirmed good predictive performance (AUC = 0.85), and the optimal cut-off value was ≥ 5 metastatic lateral lymph nodes. **Conclusions**: A substantial proportion of patients with clinically negative Level II nodes harbor occult metastasis. The number of metastatic lateral lymph nodes is an independent predictor of occult Level II involvement and may assist in tailoring the extent of lateral neck dissection in patients with PTC.

## 1. Introduction

Papillary thyroid carcinoma (PTC) is the most prevalent type of thyroid malignancy, accounting for approximately 80–85% of all thyroid cancers [1]. Although PTC is generally associated with excellent prognosis and indolent clinical course, it frequently exhibits lymphatic spread, particularly to the central compartment (level VI) of the neck. Occult lymph node metastases, defined as microscopic metastases not detected by preoperative imaging or physical examination, are reported in 20–90% of cases. While the prognostic significance of cervical lymph node metastasis on overall survival remains debatable, numerous studies have demonstrated a clear association between nodal metastasis and increased risk of locoregional recurrence [2,3]. These findings underscore the importance of accurate preoperative evaluation and appropriate surgical planning in patients with PTC.

Lateral neck metastases, including level II, require curative lymph node dissection in patients with clinically evident nodal disease. However, management of clinically negative level II lymph nodes remains a topic of ongoing debate. Routine dissection of level II in the absence of radiologic or clinical evidence may provide more accurate pathological staging and improved regional control, but it also carries the potential for substantial morbidity, particularly related to spinal accessory nerve injury, which can result in long-term shoulder dysfunction and impaired quality of life [4,5,6]. An important clinical challenge lies in the identification of patients with occult level II metastases-those not detected on preoperative imaging or physical examination but identified on postoperative pathological evaluation.

Understanding the risk factors for metastasis to clinically negative level II lymph nodes is essential for optimizing the extent of lateral neck dissection. A more selective and risk-based surgical approach may help avoid unnecessary dissection of level IIb nodes, where the morbidity risk often exceeds the likelihood of metastatic involvement. Conversely, omission of level II dissection in high-risk patients may result in residual disease and an increased risk of recurrence. Therefore, establishing refined criteria for level II dissection based on reliable clinicopathologic predictors represents a critical area of investigation in the surgical management of PTC.

This study aims to evaluate the risk factors associated with clinically negative level II cervical lymph node metastasis in patients with PTC who present with clinically positive nodes in levels III or IV. By identifying these risk factors, we aim to inform more individualized and evidence-based surgical decision-making, reduce operative morbidity, and enhance patient-specific treatment planning.

## 2. Materials and Methods

This retrospective cohort study was conducted in accordance with the Declaration of Helsinki and approved by the Institutional Review Board of Daejeon St. Mary’s Hospital, a tertiary referral center located in Daejeon, South Korea (IRB No. DC25RISI0034; approved 22 July 2025). Patient consent was waived due to the retrospective design of the study and use of anonymized medical records, with IRB approval. The study design and reporting adhered to the STROBE (Strengthening the Reporting of Observational Studies in Epidemiology) guidelines.

We reviewed the medical records of patients who underwent total thyroidectomy with therapeutic lateral neck dissection for PTC between January 2015 and December 2022. Patients were eligible for inclusion if they met all the following criteria: (1) Histologically confirmed PTC; (2) Clinically positive lateral cervical lymph node metastasis involving Level III and/or IV confirmed by preoperative imaging (ultrasound, Computed Tomography (CT)) and fine-needle aspiration cytology (FNAC); (3) Clinically negative Level II lymph nodes based on imaging and physical examination; and (4) Underwent dissection of Level II lymph nodes, regardless of clinical suspicion.

Exclusion criteria included: (1) Recurrent or persistent PTC; (2) Prior neck surgery or radiotherapy; (3) Preoperative clinically suspicious Level II lymph node metastasis; (4) Incomplete surgical or pathological data; and (5) Concurrent malignancies, such as medullary thyroid carcinoma, follicular carcinoma, or other organ cancers.

To minimize selection bias, all eligible patients who met the inclusion criteria during the study period were consecutively enrolled. Cases with incomplete clinical or pathological data were excluded from the analysis, and no data imputation was performed. No sensitivity analyses were conducted.

### 2.1. Preoperative Evaluation

All patients underwent a comprehensive preoperative evaluation including physical examination, thyroid function tests, neck ultrasonography, and contrast-enhanced neck CT. FNAC was performed on suspicious lateral cervical lymph nodes, primarily in Level III or IV. In our institution, FNAC was generally performed for thyroid nodules ≥ 10 mm, or for nodules < 10 mm when they exhibited high-suspicion sonographic features or were associated with suspicious lymphadenopathy. No clinically suspicious lymph nodes were identified at Level II on either imaging or physical examination. The clinical nodal status was classified using American Joint Committee on Cancer (AJCC) criteria as clinically node-negative (cN0) or node-positive (cN1), based on nodal size, shape, fatty hilum status, and enhancement on imaging.

### 2.2. Surgical Procedure

All surgeries were performed by board-certified endocrine surgeons experienced in thyroid and neck procedures. Total thyroidectomy and central lymph node dissection (CLND) with therapeutic lateral lymph node dissection (LLND) were performed as initial treatment. LLND routinely included Levels II, III, and IV, in accordance with American Head and Neck Society guidelines. In all cases, preservation of the spinal accessory nerve, internal jugular vein, and sternocleidomastoid muscle was prioritized unless direct tumor invasion was evident. Two patients who underwent Level V dissection were excluded. Bilateral CLND and LLND were performed in patients with bilateral lateral neck metastases, and one patient underwent bilateral LLND. In addition, all patients who underwent lateral neck dissection also received bilateral central neck dissection.

### 2.3. Pathological Assessment

All surgical specimens were evaluated by experienced pathologists following World Health Organization (WHO) guidelines. The following tumor characteristics were assessed: size, multifocality, bilaterality, and extrathyroidal extension (ETE). Lymph node status was evaluated for each neck level, including the number and maximum diameter of metastatic nodes. Occult Level II lymph node metastasis was defined as pathologically confirmed metastasis in Level II nodes that had been clinically negative on preoperative evaluation. Dissected lateral neck lymph nodes were labeled intraoperatively according to anatomical level (II, III, IV), and both the number of harvested and metastatic nodes per level were documented.

### 2.4. Data Collection and Variables

Demographic data (age, sex), tumor characteristics (size, multifocality, bilaterality, ETE), and lymph node data (location, number, and size of metastatic and harvested lymph nodes, presence of extranodal extension were extracted from electronic medical records and pathology reports. Patients were stratified into two groups based on the presence or absence of occult Level II lymph node metastasis.

### 2.5. Statistical Analysis

Descriptive statistics were used to summarize data. Continuous variables were expressed as mean ± standard deviation and compared using Student’s *t*-test or Mann–Whitney U test, depending on distribution. Categorical variables were presented as frequencies and percentages, and compared using chi-square or Fisher’s exact tests, as appropriate.

Univariate logistic regression was performed to identify variables associated with occult Level II metastasis. Variables with a *p*-value < 0.10 in univariate analysis were included in a multivariate logistic regression model using a backward stepwise selection to determine independent risk factors. Adjusted odds ratios and 95% confidence intervals were calculated. A *p*-value < 0.05 was considered statistically significant. All analyses were performed using SPSS software (version 20.0; IBM Corp., Armonk, NY, USA).

## 3. Results

A total of 1247 patients who underwent thyroidectomy for PTC between January 2015 and December 2022 were retrospectively analyzed. The mean age was 47.9 ± 12.7 years, with 477 patients (38.3%) younger than 55 years and 770 (61.7%) aged 55 years or older. The cohort was predominantly female (80.9%, *n* = 1009). The mean tumor size was 0.94 ± 0.072 cm. Multifocal tumors were present in 498 patients (39.9%), while 749 patients (60.1%) had unifocal disease. ETE was noted in 677 patients (54.3%). Central lymph node metastasis (CLNM) was found in 447 patients (35.8%). Among all patients, 1180 (94.6%) underwent CLND only, while 67 (5.4%) underwent both CLND and therapeutic LLND (Table 1).

We compared the clinicopathological characteristics of the entire cohort (*n* = 1247) based on the presence or absence of lateral lymph node metastasis (LLNM). The results of this comparison are summarized in Table 2.

No statistically significant differences were observed between patients with and without LLNM in terms of mean age (47.93 ± 11.73 vs. 46.52 ± 15.14 years, *p* = 0.458) or sex distribution (female:male = 80.8:19.2% vs. 71.6:28.4%, *p* = 0.171). Although patients under 55 years of age were more frequently observed in the LLNM-negative group compared to the LLNM-positive group (50.7% vs. 38.3%), the difference did not reach statistical significance (*p* = 0.053). In contrast, several tumor-related and nodal features showed significant associations with LLNM. The LLNM-positive group exhibited significantly larger primary tumor size (1.75 ± 1.17 cm vs. 0.94 ± 0.72 cm, *p* < 0.001) and a higher incidence of extrathyroidal extension (ETE) (86.6% vs. 54.3%, *p* < 0.001). Tumor multifocality did not differ significantly between the two groups (*p* = 0.309). Regarding central lymph node involvement, patients with LLNM demonstrated a significantly higher number of metastatic central lymph nodes (4.27 ± 3.57 vs. 2.78 ± 2.32, *p* = 0.001) and larger maximal size of metastatic central lymph nodes (1.08 ± 0.79 cm vs. 0.45 ± 0.35 cm, *p* < 0.001). Furthermore, extranodal extension was more frequently observed in the LLNM-positive group (17.9% vs. 7.4%, *p* = 0.008). Although the rate of lymphovascular invasion was higher in the LLNM-positive group (1.5% vs. 0.2%), the difference was not statistically significant (*p* = 0.244).

Among the 67 patients with pathologically confirmed LLNM, patients were stratified according to the presence of occult level II lymph node metastasis. Occult metastasis to level II was identified in 24 patients (35.8%), whereas the remaining 43 patients (64.2%) showed no pathological involvement of level II nodes despite undergoing dissection (Table 3).

There were no statistically significant differences between the groups in terms of mean age (47.33 ± 12.57 vs. 45.08 ± 19.14 years, *p* = 0.610), sex distribution (female:male = 69.8%:30.2% vs. 75.0%:25.0%, *p* = 0.780), or proportion of patients younger than 55 years (48.8% vs. 54.2%, *p* = 0.800). However, patients with occult level II metastasis had significantly larger primary tumors compared to those without (2.18 ± 1.31 cm vs. 1.51 ± 1.02 cm, *p* = 0.024). Additionally, they exhibited a significantly higher number of CLNM (5.88 ± 4.41 vs. 3.37 ± 2.66, *p* = 0.005), greater maximum size of CLNM (1.44 ± 1.07 cm vs. 0.87 ± 0.48 cm, *p* = 0.004), and a higher number of metastatic lateral lymph nodes (7.63 ± 3.75 vs. 3.19 ± 2.21, *p* < 0.001). Other tumor characteristics, such as multifocality (50.0% vs. 44.2%, *p* = 0.799), extrathyroidal extension (91.7% vs. 83.7%, *p* = 0.472), extranodal extension (25.0% vs. 14.0%, *p* = 0.324), and lymphovascular invasion (0% vs. 4.2%, *p* = 0.358), did not show statistically significant differences between the groups. These findings suggest that while several features did not differ significantly between groups, larger tumor size, greater burden of central and lateral nodal metastasis, and larger metastatic node size may serve as potential indicators of occult level II involvement.

To identify independent predictors of occult level II involvement, multivariate logistic regression analysis was performed, including variables with *p* < 0.10 in univariate analysis. Among these, only the number of metastatic lateral lymph nodes remained an independent predictor (OR = 1.57, 95% CI: 1.213–2.044, *p* = 0.001). The final multivariate logistic regression model demonstrated a Nagelkerke R^2^ of 0.46, indicating moderate explanatory power.

**Figure 1:**
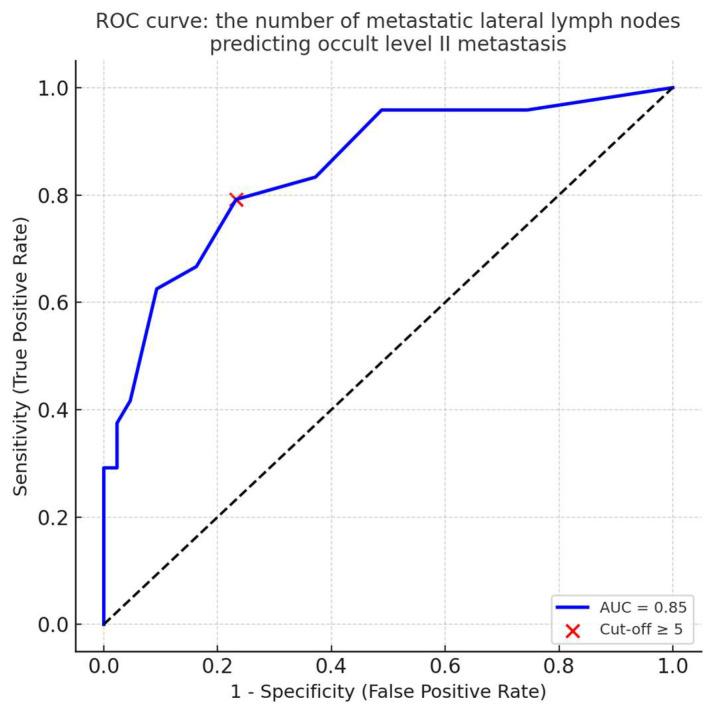
ROC curve: the number of metastatic lateral lymph nodes predicting occult level II metastasis. The curve illustrates the discriminatory ability of the number of metastatic lateral lymph nodes for predicting occult level II involvement. The red dot indicates the optimal cut-off point (≥5 nodes).

To further evaluate the predictive performance of the number of metastatic lateral lymph nodes, ROC curve analysis was performed. The ROC analysis demonstrated good discriminatory power, with an AUC of 0.85. The optimal cut-off value was identified as ≥5 metastatic lateral lymph nodes, yielding a sensitivity of 79%, a specificity of 77%, a positive predictive value (PPV) of 66%, and a negative predictive value (NPV) of 87% (Figure 1).

This indicates that each additional metastatic lateral lymph node increases the odds of occult level II metastasis by 57%. Although the size of metastatic central lymph node showed a trend toward significance (OR = 3.71, 95% CI: 0.918–15.010, *p* = 0.066), it did not reach statistical significance. Tumor size (OR = 1.03, *p* = 0.916) and number of metastatic central lymph node (OR = 1.17, *p* = 0.207) were also not identified as independent predictors (Table 4).

## 4. Discussion

In this study, we comprehensively evaluated the clinicopathological features associated with LLNM and occult level II metastasis in patients with PTC. Our findings provide meaningful insights into the distribution patterns and risk factors of cervical lymph node involvement, particularly in the lateral compartment, and underscore their relevance in guiding surgical decision-making and postoperative oncologic surveillance.

Despite the overall favorable prognosis of PTC, the presence of cervical lymph node metastasis—particularly within the lateral neck compartment—is associated with an increased risk of locoregional recurrence and may significantly impact the extent of surgical intervention and the consideration for adjuvant therapy [7,8]. While aggressive surgical management is often employed in patients with nodal involvement, the extent of lymph node dissection must be judiciously determined due to the potential for postoperative morbidity. Notably, extensive lateral neck dissection increases the risk of injury to the spinal accessory nerve, potentially resulting in long-term shoulder dysfunction and decreased quality of life. Thus, careful balancing of oncologic benefit and surgical morbidity is essential when planning the extent of lateral neck dissection.

Recent studies have indicated that preoperative ultrasonography can improve the assessment of metastatic involvement, particularly in the lateral neck compartment, thereby enabling more tailored and selective surgical strategies [9,10]. In our cohort, LLNM was significantly associated with markers of aggressive tumor behavior. Patients with LLNM demonstrated larger primary tumor size, a higher incidence of ETE, and a greater number and size of CLNM, as detailed in Table 2. These findings align with previous research suggesting that tumor burden and invasive potential are key determinants of lateral lymphatic spread [11,12]. Notably, tumor multifocality and patient sex were not significantly correlated with LLNM, reinforcing the notion that intrinsic tumor aggressiveness—rather than the number of tumor foci or patient demographics—is more predictive of metastatic behavior.

The role of prophylactic lateral neck dissection remains contentious, as its impact on locoregional control and overall survival in PTC has not been definitively demonstrated. Additionally, the clinical relevance of occult lymph node metastasis continues to be a subject of debate [13]. In our study, the average number of metastatic lymph nodes identified in level II was 0.7, with an occult metastasis rate of 35.8%.

This suggests that while overt tumor burden in level II may appear limited, microscopic disease is relatively common. Importantly, these occult metastases were typically of small volume and undetectable by preoperative imaging, indicating a low disease burden. In such cases, the therapeutic benefit of routine level II dissection must be weighed against the potential for increased surgical morbidity. Given the morbidity associated with extensive lateral neck dissection—particularly at level II, which poses a higher risk for spinal accessory nerve-related complications [14,15]—a selective approach may be justified. Specifically, small-volume occult metastases might be adequately addressed through postoperative radioactive iodine (RAI) therapy, thereby avoiding the need for prophylactic dissection in patients without high-risk features. This tailored strategy may offer a balance between oncologic safety and quality of life preservation.

A key focus of this study was to evaluate occult level II metastasis—defined as metastases that were clinically and radiologically undetectable preoperatively but confirmed through pathological analysis after surgery. In our cohort, 35.8% of patients with LLNM harbored occult metastases in level II lymph nodes. These patients were characterized by more aggressive disease profiles, including significantly larger primary tumors, greater numbers and larger sizes of CLNM, and a higher burden of LLNM. These findings underscore the diagnostic limitations of preoperative imaging, which often underestimates the extent of nodal involvement, particularly in higher cervical levels such as level II [16,17]. As a result, relying solely on imaging for surgical planning may lead to under-treatment in a considerable subset of patients.

From a surgical standpoint, the decision to perform level II dissection in patients without radiological evidence of metastasis remains a subject of debate. While routine inclusion of level II may improve locoregional control, it also increases the risk of complications-particularly injury to the spinal accessory nerve, which can lead to persistent shoulder dysfunction and impaired quality of life [5,18]. Conversely, omitting level II dissection in patients with substantial nodal burden may result in residual microscopic disease, undermining long-term oncologic outcomes. Our multivariate logistic regression analysis (Table 4) identified the number of metastatic lateral lymph nodes as the only independent predictor of occult level II metastasis (OR = 1.57, *p* = 0.001), suggesting that intraoperative assessment of lateral nodal burden could serve as a useful guide for surgical decision-making. Additionally, the size of CLNM showed a trend toward significance (*p* = 0.066), indicating that both central and lateral nodal characteristics may be relevant in estimating the risk of occult metastasis and warrant consideration during operative planning.

Previous studies have suggested various clinicopathological features—such as tumor size, ETE, and the number of metastatic central lymph node—as predictors of occult nodal involvement [19,20]. Our results reinforce the significance of the lateral nodal burden, which emerged as a more reliable predictor of occult level II metastasis compared to central compartment factors. Notably, the relatively high incidence of occult level II disease (35.8%) in our cohort underscores the need for heightened vigilance in patients with extensive lateral nodal metastasis. In particular, the presence of ≥4 metastatic lateral lymph nodes or bulky CLNM may warrant consideration for level II dissection, even in the absence of radiologic suspicion. However, the accuracy of preoperative imaging can vary depending on radiologist expertise, and subtle metastatic deposits may be overlooked. Furthermore, interobserver variability in pathological assessment may impact the detection of microscopic disease, contributing to potential underestimation of nodal involvement.

Our baseline cohort (Table 1) largely reflects the typical demographic and pathological features of PTC, including a strong female predominance, high rates of multifocality, and extrathyroidal extension. These characteristics offer a representative foundation for risk stratification and predictive model development. Notably, only 5.4% of patients in the entire cohort underwent therapeutic LLND, highlighting that lateral neck dissection is generally reserved for cases with clear clinical or radiologic suspicion. This low rate of dissection raises the possibility that occult lateral disease, including at level II, may be underrecognized and potentially undertreated in routine clinical practice.

This study has several limitations. First, the retrospective design and single-center setting may introduce selection bias and limit the generalizability of our findings to broader patient populations. Second, the relatively small number of patients with pathologically confirmed occult level II metastasis may have reduced the statistical power of our multivariate analysis and the robustness of predictive associations. Third, long-term oncologic outcomes, including recurrence rates and disease-specific survival, were not evaluated in this study and should be investigated in future prospective research to validate the clinical relevance of our findings. Fourth, potentially relevant clinical and biological factors, such as body mass index (BMI), dietary habits, and hematological inflammatory scores, were not available in our dataset and therefore could not be assessed [21].

## 5. Conclusions

In summary, this study highlights important clinicopathological factors associated with lateral lymph node metastasis and occult level II involvement in patients with papillary thyroid carcinoma. Among these, the number of metastatic lateral lymph nodes emerged as the most significant independent predictor of occult level II metastasis. Given the potential for residual disease in patients with substantial lateral nodal burden, careful intraoperative assessment is warranted. Surgeons should consider extending the dissection to level II in selected high-risk cases, even when preoperative imaging does not suggest involvement. These findings underscore the need for a risk-adapted surgical approach, balancing oncologic completeness with the goal of minimizing unnecessary morbidity in the management of PTC.

## Figures and Tables

**Table 1:** Baseline clinicopathological characteristics of the study population (*n* = 1247).

Variable	*n* (%) or Mean ± SD
Age (year)	47.9 ± 12.7
<55	477 (38.3)
≥55	770 (61.7)
Sex	
Male	238 (19.1)
Female	1009 (80.9)
Tumor size (cm)	0.94 ± 0.072
Multi-focality	
Present	498 (39.9)
Absent	749 (60.1)
ETE	
Present	677 (54.3)
Absent	570 (45.7)
CLNM	
Present	447 (35.8)
Absent	800 (64.2)
Neck dissections	
CLND without LLND	1180 (94.6)
CLND with LLND	67 (5.4)

Values are presented as number (%), unless otherwise indicated. CLND: central lymph node dissection; CLNM: central lymph node metastasis; ETE: extrathyroidal extension; LLND: lateral lymph node dissection; SD: standard deviation.

**Table 2:** Comparison of clinicopathological features between patients with and without LLNM.

	LLNM (−)	LLNM (+)	*p* Value
Patient characteristics			
Age (year)	47.93 ± 11.73	46.52 ± 15.14	0.458
Age (<55 years:≥55 years, %)	50.7:49.3	38.3:61.7	0.053
Sex (Female:Male,%)	80.8:19.2	71.6:28.4	0.171
Tumor characteristics			
Tumor size (cm)	0.94 ± 0.72	1.75 ± 1.17	<0.001
Multi-focality (+:−, %)	39.9:60.1	46.3:53.7	0.309
ETE (+:−, %)	54.3:45.7	86.6:13.4	<0.001
CLN status			
Number of metastatic CLN (*n*)	2.78 ± 2.32	4.27 ± 3.57	0.001
Maximal size of metastatic CLN (cm)	0.45 ± 0.35	1.08 ± 0.79	<0.001
Other pathological features			
Lymphovascular invasion (+:−, %)	0.2:99.8	1.5:98.5	0.244
Extra-nodal extension (+:−, %)	7.40:92.6	17.9:82.10	0.008

Values are presented as mean ± SD or %, as appropriate. CLN: central lymph node; ETE: extrathyroidal extension; LLNM: Lateral lymph node metastasis.

**Table 3:** Clinicopathological comparison between patients with and without occult Level II lymph node metastasis among those with LLNM.

	Occult Level II Metastasis (−)	Occult Level II Metastasis (+)	*p* Value
*n* (%)	43 (64.2%)	24 (35.8%)	
Patient characteristics			
Age (mean ± SD)	47.33 ± 12.57	45.08 ± 19.14	0.610
Age (<55 years:≥55 years, %)	48.8:51.2	54.2:45.8	0.800
Sex(F:M %)	69.8:30.2	75.0:25.0	0.780
Tumor characteristics			
Tumor size (cm)	1.51 ± 1.02	2.18 ± 1.31	0.024
Multi-focality (+:−, %)	44.2:55.8	50.0:50.0	0.799
ETE (+:−, %)	83.7:16.3	91.7:8.3	0.472
Status of LN metastasis			
Number of metastatic CLN (*n*)	3.37 ± 2.66	5.88 ± 4.41	0.005
Size of metastatic CLN (Max, cm)	0.87 ± 0.48	1.44 ± 1.07	0.004
Extra-nodal extension (+:−, %)	14.0:86.0	25.0:75.0	0.324
Lympho-vascular invasion (+:−, %)	4.2:95.8	0.0:100	0.358
Metastatic LLN (*n*)	3.19 ± 2.21	7.63 ± 3.75	<0.001

Values are presented as mean ± SD or % as indicated. Occult Level II metastasis was defined as pathologically confirmed metastasis not detected by preoperative imaging. ETE: extra thyroidal extension; CLN: central lymph node; LLN: lateral lymph node.

**Table 4:** Multivariate logistic regression analysis of predictors for occult level II lymph node metastasis in patients with clinically negative level II nodes.

	OR	95% CI	*p* Value
Tumor size (cm)	1.03	0.543–1.973	0.916
Number of metastatic CLN	1.17	0.918–1.481	0.207
Size of metastatic CLN (cm)	3.71	0.918–15.010	0.066
Number of metastatic LLN	1.57	1.213–2.044	0.001

Variables included in the model were those with *p* < 0.10 in univariate analysis. Occult level II metastasis was defined as pathologically confirmed metastasis in clinically negative level II nodes. CI: confidence interval; CLN: central lymph node; LLN: lateral lymph node; OR: odds ratio.

## Data Availability

The datasets used and/or analyzed during the current study are available from the corresponding author on reasonable request.

## Abbreviations

The following abbreviations are used in this manuscript:

CT	Computed Tomography
CLND	Central lymph node dissection
CLNM	Central lymph node metastasis
FNAC	Fine-needle aspiration cytology
ETE	Extrathyroidal extension
LLND	Lateral lymph node dissection
LLNM	Lateral lymph node metastasis
PTC	Papillary thyroid cancer
